# Memento mori

**DOI:** 10.1017/S1478951525000203

**Published:** 2025-03-07

**Authors:** Ellen Zhang

**Affiliations:** Department of Medicine, Stanford Health Care, Stanford, CA, USA

- I) before

feathers slick/cranes arch necks/take off with our imprinted gazes/our skin unfolds in light/nutrition of wildflowers/algae floats steady/blooming/soft edges glistens blue-green/mossy tendrils/inhale sun/odor of wetness and damp/cacophony of birds/calling of secrets looming in shape/roots stirring beneath floor/dusk hiding regret

- II) during

rustling bedsheets/ache of ribs/sharp inhale/amplified/our bodies hum/things we do not hold/chickadee flutter/hollow bones/swaying sinew/sagging flesh/unfolding guilt/our hands grasp each other’s/pinkies link/linger/solidifying shape of your knuckles/veins traced as marked/estuary of sharp blue/inside/sterile cold with white linoleum/outside/dust rise/yellow/dusk sets

- III) after

voices at eulogies/quivering like sparrow wings/lost in an airport/map without edges/earth shrugged/weight of black/saturated heat/on pavement/rising/peeling away words/of days/every summer I migrate back/softening my vertebrae against bark/fold inside branching hours/squint into/sun/let me take and take only/touch the soil/filled with minerals and rust/decay and renewal/take it in/dusk repeats

